# Intrahousehold Inequalities in Child Rights and Well-Being. A Barrier to Progress?

**DOI:** 10.1016/j.worlddev.2016.02.005

**Published:** 2016-07

**Authors:** Laura Rodríguez

**Affiliations:** The University of Manchester, UK

**Keywords:** intrahousehold, inequality, children, multidimensional well-being, decomposition

## Abstract

This paper uses UNICEF’s Multiple Indicators Cluster Surveys cross-country data to shed light on unequal investments in the wellbeing of children (boys and girls) within the household. The paper proposes a new methodology to measure the extent of gender intrahousehold inequality and its contribution to overall levels of inequality using an L-Theil index decomposition. The individual and joint distribution of inequality in four key indicators of child wellbeing is analyzed: stunting, birth registration, school attendance, and time spent on work and chores (working hours) in the search for evidence of gender bias. Evidence from various separate county studies had shown that the direction of the gender bias is not universal. Such conclusion holds when using a consistent methodology and comparable cross-country datasets; this paper shows that disparities inside households do not follow the same bias toward one or the other gender in all countries and the direction of the bias is not the same across indicators of wellbeing. While progress in improving child wellbeing has occurred in many countries, inequalities remain. Intrahousehold inequalities might still be considered a priority in an agenda focused on closing these progress gaps.

## Highlights

• Gender intrahousehold inequality is measured with an L-Theil index decomposition.
• Inequality in multidimensional child wellbeing is measured with four indicators.
• Disparities inside households do not follow a consistent gender bias in all countries.
• The size and direction of these disparities also vary across wellbeing indicators.

## 1 Introduction

Non-unitary models of household behavior—where households do not have a unique set of preferences and are not assumed to jointly maximize some household welfare function—have long been acknowledged in the economic literature. Collective models of household behavior, that explicitly incorporate the interactions that occur within households in the determination of the internal resource allocation, are useful to explain the presence of different outcomes for different household members, particularly children and along gender lines. Still, many empirical measures of wellbeing have treated households as if their members enjoy an equal share of all household resources. For analytical convenience, most policy analysis assumes that, within households, individual wellbeing is the adult-equivalent average of the household to which the individual belongs; this can lead to an underestimation of overall poverty and inequality (Haddad & Kanbur, 1990). When household resources—whether money, consumption goods, or investments—are not equally distributed among household members, particular individuals may be worse off than others, and could effectively be in poverty, even when household averages indicate the contrary. In terms of child wellbeing, the neglect of intrahousehold inequalities conceals the outcomes for those children who fare below their household average, affecting the assessment of the levels and trends of child poverty. This paper attempts to measure the extent of gender inequality within households and to show how it contributes to overall inequality in child outcomes.

Examining and tackling the differences that occur within households is important for ensuring children’s wellbeing and the realization of their rights. Unequal household investments in children tend to carry over into adulthood. Although other factors can still affect wellbeing over the life-course, systematic biases against boys or girls during childhood are linked to poverty traps and to the intergenerational transmission of poverty (Bhalotra and Rawlings, 2011, Harper et al., 2003).

Girls and women are believed to bear a heavy share of the burden of poverty, yet good data and detailed analysis for a wide range of countries are needed to corroborate this claim (Marcoux, 1998). Preferential treatment of some children is evident in many societies, resulting in unequal outcomes in child development with life-long implications. Patterns of bias in favor of boys or girls, however, differ across wellbeing indicators and countries. For instance, biases in land and productive asset inheritance have been found to favor boys (Bird, 2011, Cooper, 2011, Doss et al., 2011, Estudillo et al., 2001), while girls have relatively low survival rates in Asia (Klasen, 2008, Sen, 1992)^1^ and perhaps in Africa as well (Klasen, 1996). Despite expansion in general education, they still also have lower education participation rates in India (Azam & Kingdon, 2013), and are subject to lower parental aspirations in India and Ethiopia (Dercon & Singh, 2013). However, this last study also found that in the other two countries analyzed, Peru and Vietnam, the bias ran in the opposite direction. Similarly, nutrition indicators show a bias against boys, especially for younger children in Sub-Saharan Africa (Sahn and Stifel, 2002, Svedberg, 1990), and also in India (Andhra Pradesh), Ethiopia, Peru, and Vietnam (Dercon & Singh, 2013). At the same time, nutrition indicators have also been found to be biased against girls in some South Asian countries (e.g., for India, see Deaton, 1989, Sen, 1988, Sen and Sengupta, 1983; for Bangladesh, see L. C. Chen, Huq, and D’Souza (1981)), highlighting that the direction of the bias can vary across different countries. Among the mechanisms that have been singled out as leading to intrahousehold inequalities are those that affect the bargaining power of the household decision makers. In particular, those affecting mothers’ bargaining position have been shown to be highly relevant, perhaps because there is some evidence that female-headed households prioritize investments in children to a greater extent than households headed by men (Chant, 2007). Women’s bargaining position can improve through higher employment rates (Mammen and Paxson, 2000, Rosenzweig and Schultz, 1982), command over productive resources (Udry, 1996), or even as a result of men’s migration (Chen, 2013). The expected return of investing in girls, as well as the opportunity cost for households also play an important role (Song, Appleton, & Knight, 2006).

Despite this wide range of country evidence on gender biases, systematic evaluations of cross-country evidence of the extent and the direction of the bias of intrahousehold inequality in child wellbeing are uncommon. In addition, inequalities in different dimensions may balance each other out, for example when parents compensate underinvestment in one area with overinvestment in another. Estudillo *et al.* (2001) for instance, found in the Philippines, that parents compensate lower inheritance transfers of land with higher investments in schooling for girls, resulting in very little difference in lifetime incomes between sons and daughters. A multidimensional approach to the measurement of inequalities in child wellbeing is necessary to gain a fuller understanding of these biases and is an important aspect of diagnosing the barriers to progress.

The aim of this paper is to use existing cross-country data to shed light on unequal investments in the wellbeing of children (boys and girls) within the household. The individual and joint distribution of inequality in four key indicators of child wellbeing is analyzed: stunting, birth registration, school attendance, and time spent on work and chores (working hours). Knowing more about inequalities inside households, as well as about inequalities occurring across multiple aspects of wellbeing would be of great value to enhance our understanding of the magnitude and nature of child poverty and gender inequality. The next section briefly reviews the key measurement issues that this article engages with, namely the measurement of multidimensional wellbeing, of multidimensional inequality, and of intrahousehold inequality. Section 3 presents the methodological approach situated in this literature. Section 4 presents the results of the analysis for 20 developing countries. The final section discusses some of the implications of these results and avenues for future research.

## 2 Measurement issues

### (a) Multidimensional child wellbeing

The first point of departure for this study is an interest in measuring inequality in child wellbeing from a multidimensional perspective. New ground was broken in the measurement of child poverty and wellbeing with UNICEF’s ‘Global Study on Child Poverty and Disparities’ (UNICEF, 2007), which combined the household income poverty measure with the multidimensional Bristol deprivations approach (Gordon, Nandy, Pantazis, Pemberton, & Townsend, 2003), the methodology used to produce the first internationally comparable estimates of child poverty across a large number of developing countries.^2^ Although it captured the multidimensionality of child deprivation and was useful for analyzing disparities across countries, this study adopted a household-level approach to measurement, which could mask disparities within households, and thus not suitable for an intrahousehold inequality analysis.

The use of household-level data not only conceals differences between household members, particularly children, but also poses an additional problem: if child poverty is made equivalent to overall household poverty, policy responses may address the main underlying causes of poverty but fail to account for child-specific concerns and experiences as well as for intrahousehold inequalities. In the multidimensional poverty context, Vijaya, Lahoti, and Swaminathan (2014) show how this matters. In their Indian illustration, gender differences in poverty are virtually non-existent when individuals are assigned their household poverty status, but they are large when individual-level information is used to define poverty. Furthermore, they show how many poor men and women can live in non-poor households.

A crucial discussion then concerns the space in which to measure gender inequalities in child wellbeing. Some spaces may be more problematic than others. For example, measuring inequality in income poverty may be suitable for comparing households but less so for capturing intrahousehold distributions. A monetary metric would be even more unfit for the focus on children. This paper measures inequality in a multidimensional space. This follows the multidimensional definition of child wellbeing set by the 1990 Convention on the Rights of Child (CRC) and the tradition of child wellbeing studies since the aforementioned UNICEF and Bristol studies. A key difference arises from the concern for intrahousehold inequality, which requires the measurement to be carried at the individual level.

### (b) Multidimensional inequality

A key issue in the debates over how to measure gender inequality is whether composite indicators add value (Klasen, 2007). This is especially relevant in the multidimensional case where the consideration of the correlation between the various dimensions is important in the analysis. Some authors have restricted the analysis to each of the individual distributions of the dimensions of wellbeing, without regard to its correlation with other dimensions. This approach is widely used by studies focused on non-income inequalities, particularly health and education (e.g., Gakidou and King (2002) in health; Thomas, Wang, and Fan (1999) and Checchi (2000) in education; and Sahn and Younger (2006) in both health and education). Others have attempted to aggregate the various dimensions into a uni-dimensional index of deprivation and then analyze its distribution for different sub-groups. The Alkire–Foster counting method (Alkire & Foster, 2011), applied in Roche’s (2013) study of child poverty in Bangladesh and in UNICEF’s Multiple Overlapping Deprivation Analysis (MODA) to construct an aggregate deprivation index using the corresponding dimensions outlined in the Bristol approach, are examples of such approach. Although these indices were developed to measure child deprivation, the resulting aggregate index can be used to measure disparities using a traditional Generalized Entropy (GE) measure, for example, or analyzing how the index is distributed across regions or population sub-groups. Still, the weighting in such multidimensional indices remains a contested issue (Decancq & Lugo, 2013), and thus aggregating can obscure the relationships between the different dimensions. Moreover, a composite index has a further limitation from an empirical stand. It requires that data to be available for each individual on all the dimensions of wellbeing. These can be difficult to obtain at the individual level in the case of child wellbeing, especially because some dimensions are only relevant and measured for certain age groups.

An intermediate approach considers the correlation between dimensions explicitly but without aggregating them. A well known example of this type of analysis is Wagstaff’s (2002) study of health inequality in which he defines a concentration curve of health outcomes ranked across socioeconomic quintiles. If the curve coincided with the diagonal or line of equality, it was concluded that all children irrespective of their socioeconomic status enjoyed the same health outcomes. As pointed out by Sahn and Younger (2006), the problem of this approach is that it gives primacy to income above the other dimensions of wellbeing by ordering the distribution by socioeconomic categories; inequalities in other dimensions are only relevant if they are correlated with socioeconomic inequality. A way to avoid the income primacy is to compute distributional measures across the full set of pairwise combinations of dimensions.

### (c) Intrahousehold inequality

Section 2(a) emphasized the importance of an individual-level approach to the assessment of poverty and wellbeing. A stronger focus on individual child outcomes is more appropriate for capturing disparities in child poverty and more useful for addressing the protection of child rights (Fajth & Holland, 2007). The case is stronger when considering inequality within households. Measuring the distribution of resources or outcomes within households should be a relatively straightforward task in the presence of individual-level data. This would allow to directly track differences between boys and girls. For instance, the Gender Parity Index used in the Women’s Empowerment in Agriculture Index (Alkire, Meinzen-Dick, Peterman, Quisumbing, Seymour, & Vaz, 2013) computes the gap between outcomes for women and men in each household to get a sense of the shortfall between genders. However, data availability often limits such an approach and different alternatives have been used to surmount this difficulty.

A first data limitation, is the absence of individual-level data. Deaton (1989), for example, approximated individual budget allocations to boys and girls using non-child expenditures (i.e., tobacco, alcohol, and adult clothing). Compared to childless households, one would expect a reduction in the income available for non-child expenditures in households with children. If this reduction were systematically larger in households with male children than in those with female children, it would suggest that households were diverting more resources to the male children. The present study limits the analysis to indicators that can be directly measured at the individual level, but addresses a second data limitation.

Many indicators used to capture the different dimensions of child wellbeing are not cardinal but instead are either ordinal or binary (for example indicating whether a child is undernourished or not, attends school or not, or has been vaccinated or not). In this case, even if individual-level data are available, gender inequality cannot be computed as the gap in the outcomes of boys and girls. In this case, it is common to use a regression in which the different outcomes are regressed on a gender dummy. Dercon and Singh (2013) used this approach to measure inequalities in child nutrition, educational achievements, educational aspirations, subjective wellbeing, and psychological competencies. First, they compared the average achievements between girls and boys at various ages and second, they regressed outcomes on a gender dummy and some household characteristics (i.e., total consumption expenditure, education of the mother, household size, ethnicity/caste, and urban/rural location). The significance and direction of the gender dummy indicated the presence of gender inequality. Quisumbing (1994) also used a similar measure to analyze parental decisions about inheritance and child education investments in the Philippines, adding family fixed-effects as an attempt to capture differences in siblings within the same family.

Finally, the interest in this paper is to show the contribution of intrahousehold inequality to overall inequality in child wellbeing. A method which is relevant to capture not only the direction of the bias but also the extent of intrahousehold inequality, is to use a summary inequality measure such as the Gini coefficient or the General Entropy (GE) measures. The advantage is that an aggregate inequality index can be broken down into two components: within-household and between-household inequality, allowing to distinguish the extent of inequality occurring within and between households respectively. Sahn and Younger (2009) used this to measure gender differences in the standard of living of adults. Using Body Mass Index, they constructed a household-specific L-Theil Index and measured within- and between-household inequality using the decomposability property of the General Entropy (GE) indices. Their findings show that at least 55% of overall inequality in the seven countries examined can be attributed to the within-household component. However, such measures require cardinal data for their computation which typically limits the analysis to cardinal measures of wellbeing such as income, consumption, or anthropometric indicators as in the study of Sahn and Younger (2009).

## 3 Methodology

In this paper, intrahousehold inequality is presented in two ways. The first is the share of households with a gender bias: that is, households that display higher outcomes for either boys or girls. This is derived from household ratios of the achievement of girls to that of boys in each of the indicators. A ratio of one indicates complete parity; ratios greater than one indicate that girls’ achievements are higher than boys’ achievements, and vice versa for ratios lower than one. A bias for girls is evident when girls have more favorable outcomes than boys (i.e., a lower share of them are stunted or work less hours, or a higher share of them are registered at birth or attend school).^3^ This, however, only shows gender differences in each household, or the average gender differences across the country; it does not show the extent of intrahousehold inequalities in total inequalities. Here, an aggregate measure of inequality—the Theil index—is used to capture these magnitudes.

This paper follows Sahn and Younger’s (2009) approach to measuring inequality by breaking up a total inequality index into its within- and between-group components, using households as the defining groups. The innovation consists of adapting the methodology for a greater number of indicators, ordinal as well as cardinal, and thus allowing for a broader understanding of inequality in multiple dimensions of child wellbeing.

The method used is to obtain two cardinal values for each household out of the original binary indicators, so that an inequality index can be applied and then de-constructed to assess the contribution of its components, particularly to capture the share of within-household inequality.

Binary variables are recalculated as the share of girls and boys within a household above a certain threshold.^4^ Thresholds are defined following international standards set by UNICEF’s guidance on Indicators for Global Reporting (Appendix 1). For each household,

(1) $${\overset{\sim }{y}}_{g}=\frac{n_{g}}{N_{g}}$$

where $\overset{\sim }{y}$ is the reconstructed binary variable for *g* = (boys, girls), *n* is the number of individuals above/below the deprivation threshold and *N* the total number of individuals.

The units of analysis are the girls or boys *within a household*, so the outcome of this transformation is two household-level variables separately representing the outcomes of girls and boys *in each household.*^5^ In the case of stunting, for example, two observations are noted for each household: one corresponding to the share of girls who are stunted and the other to the share of boys who are stunted. For the work time indicator, which is continuous, there is no set threshold and the reconstructed household variable expresses the average number of hours worked by girls and boys in each household.

Only households that have at least one boy and one girl are kept in the sample for analysis, limiting the number of observations and reducing the sample considerably.^6^ A possible limitation of this approach is that the inequality measure does not control for the original size of the groups, in this case households. This may limit the comparability of the measure across countries, where the average household size varies^7^—but it is not clear that this results in any systematic bias.^8^ Although the implications for the measurement of inequality require further investigation, this still bypasses the main problem of measuring inequality using non-cardinal indicators and allows for the examination of inequality in multiple dimensions of wellbeing.^9^

This method proves useful for the gender analysis but it is not able to account for other biases that may occur simultaneously inside households. An important one relates to age. Because child age patterns within each household may affect the level of intrahousehold inequality—for example, older children are likely to work more hours—indicators are ‘cleaned’ of the age effect before using them for the analysis. This would result in a more accurate reflection of gender differences. In a logit regression that uses each outcome as the dependent variable and the age of the child as the only independent variable, the residual—the part that is not explained by age—is used as the clean indicator.^10^

With the household-level recalculated variables, a GE index can be computed for each indicator. This study uses an L-Theil index (GE(0)) or mean log deviation, which is a summary measure of the difference between the (natural logarithm of the) shares of the wellbeing measure and the shares of population. It reflects the extent to which the distribution of wellbeing between groups differs from the distribution of the population in those groups. When all the groups have a share of wellbeing equal to their population share, the distribution is completely equal (the overall Theil index is zero). It also gives a higher weight to the lower end of the distribution, giving higher relevance to those who are more deprived, and is sub-group decomposable. Equation (2) shows the decomposition of the L-Theil index. *N* is the entire sample size, *N_j_* is the sample size in each household *j*, $\overset{\sim }{y}=Y/N$ is the average score of the variable for the entire sample, *y_j_* is the average for household *j*, and *L_j_* is the inequality (mean log deviation) of each household *j*. The first term corresponds to the within-group component and the second, to the between-group component. Following Sahn and Younger (2009), each household represents a group. Given the transformation of the indicators given by Eqn. (1), each household is in turn represented by two observations—one for boys and one for girls. In the decomposition of the Theil index, the within-group component reveals how much of the inequality can be attributed to inequalities inside the household. When there is no such inequality across household members, the contribution of the within-group component is null. Households with no inequality within can still contribute to the between-group component if their mean outcomes differ from the mean outcome of the country as a whole.

(2) $$\mathrm{GE}(0)=\frac{1}{N}\overset{N}{\underset{i-1}{\sum }}\ln\frac{\overset{\sim }{y}}{Y_{i}}=\underset{j}{\sum }\frac{N_{j}}{N}L_{j}+\underset{j}{\sum }\frac{N_{j}}{N}\ln\left(\frac{\overset{\sim }{y}}{y_{j}}\right)$$

Because the Theil index is unbounded and depends on the unit of measurement, it is difficult to interpret in absolute terms and to make meaningful comparisons of inequality levels across variables measured in different units as is the case in child wellbeing. On the other hand, a Gini coefficient, which ranges from zero to one, gives an indication of the extent of overall levels of inequality, placing higher weight to the middle of the distribution. However, unlike the Theil index, the Gini coefficient is not perfectly decomposable,^11^ impeding the assessment of the share of inequality within households. For this reason, the Theil index, rather than the Gini coefficient, is the main measure of inequality used in this study, although the latter is also presented to give a sense of the level of overall inequality for each indicator.

Inequality measures and corresponding standard errors are computed taking into account sample design, using the sample weights designed and incorporated into each survey by UNICEF. Computations are made with the Distributive Analysis Stata Package (DASP) (Araar & Duclos, 2013) in Stata/SE V.12, which allows the sample design to be included in the estimation of standard errors. A standard *t*-test is used to assess the statistical significance of the changes in inequality and its components across the two periods. A test of proportions (*F*-test with a 95% significance level) is used to assess the difference between the shares of households favoring boys or girls for each indicator.

Data are obtained from UNICEF’s Multiple Indicators Cluster Surveys (MICS). As in the Bristol approach, the dimensions relevant to measuring child wellbeing in this study are defined drawing from the CRC. The dimensions analyzed are restricted to those that can be measured at the individual level and for boys and girls separately in MICS surveys. Some indicators are measured at the individual level, but only for one child in the household, rendering them insufficient for analysis. This has some data shortcomings: of the 17 dimensions of child wellbeing in the CRC, data constraints restrict this study’s analysis to only four of them: nutrition, education, birth registration/nationality, and some components of leisure and child labor. These indicators are measured only for children of a relevant age range, following UNICEF’s standards for global reporting (Appendix 1 shows these age ranges and operational definition of the indicators). Table 1 expands the table presented by UNICEF’s CC-MODA methodology to analyze child deprivations (De Neubourg, Chai, de Milliano, & Plavgo, 2012, p. 9) with information relevant to this study. Other indicators that could point to gender inequalities such as the sex ratio at birth, infant/child mortality, or the prevalence of child marriage are not available consistently through MICS surveys or cannot be measured at the individual level (as is the case of child mortality).^12^ Inequality is measured for two periods in time. The two latest surveys available for each country are used, corresponding roughly to a five-year distance between surveys (2000 and 2005–06 or 2005–06 and 2010–11). The actual period depends on the specific surveys available for each country.

**Table 1:** Child wellbeing dimensions, indicators, and data availability

Categories	Dimensions	CRC article no.	Indicators available	No. countries analyzed
Survival	Food nutrition	24	Stunting and underweight	15
	Water	24	No⁎	
	Health care	24	Immunization (DPT)****	
	Shelter, housing	27	No⁎	
	Environment, pollution	24	No	

Development	Education	28	School attendance and support for learning⁎⁎⁎	18
	Leisure	31	House work and chores	12
	Cultural activities	31	No	
	Information	13, 17	No⁎	

Protection	Exploitation, child labor	32	House work and chores	
	Other forms of exploitation	33–36	Female genital mutilation⁎⁎⁎	
	Cruelty, violence	19, 37	Child discipline⁎⁎⁎	
	Violence at school	28	No	
	Social security	16, 26, 27	No	

Participation	Birth registration/nationality	7, 8	Birth registration	19
	Information	13, 17	No⁎	
	Freedom of expression, views, opinion; being heard; freedom of association	12–15	No	

*Source:* Adapted from De Neubourg *et al.* (2012, p. 9) and author’s assessment.

⁎ Indicators for water and sanitation, information, and shelter are measured at the household level.

⁎⁎⁎ Indicator available in the Multiple Indicator Cluster Surveys (MICS) for some countries but not suited for the current analysis.

**** Indicator available in MICS but excluded from this analysis due to different immunization schedules in different countries, which makes it difficult to use for comparative purposes.

While the data source restricts the dimensions of child wellbeing that it is possible to analyze, MICS surveys are a comprehensive and comparable cross-country data source for developing countries and is consequently a good starting point for systematically measuring inequalities in multidimensional child wellbeing across developing countries. In fact, alongside Demographic and Health Surveys (DHS), MICS are used at the global level to track the achievement of the child-related indicators of the global development goals. A total of 20 countries are available to be analyzed (see Appendix 2 for details) but not all indicators are available for all countries or years. For each country, indicators are analyzed only if present in both periods (Table 1). The methodology presented in the paper could be applied to more detailed national-level datasets in future applications.

Children can be deprived in one or many of the dimensions of wellbeing and thus inequality can be measured individually or jointly. As a first instance, this paper analyzes the distribution of each dimension separately, opting for a dashboard approach to the measurement of inequality. In addition, it also aims to find systematic patterns of disadvantage, and thus it needs to look at the joint distribution of inequalities. The paper takes into consideration possible correlations between the various dimensions of welfare by considering joint distributions of the dimensions of wellbeing to see whether there is a systematic gender bias, but without integrating them into a single index.

A measure of association (*P* statistic) for each pairing of indicators (e.g., stunting-birth registration, stunting-school attendance, etc.) is calculated using the household ratios of achievement of girls to boys. These ratios show whether there is a bias against boys or girls or none in each household. Given that the sample size is reduced with each additional indicator,^13^ it is not possible to analyze joint distributions for combinations of three or all four indicators at the same time.

As an illustration of the *P statistic* consider the cross-tabulation of stunting and birth registration for the whole sample of countries in Table 2.^14^ Out of all 27,421 households, 4,597 have a bias for girls in both stunting and birth registration. This corresponds to a relatively small proportion (17%) of the total number of households in the sample, but it is a very large proportion (71%) of the total possible ‘match’ cases—that is, the households where there is a bias for girls (6,442 households in this example). The *P* statistic captures this relationship. Because some of the indicators are only relevant and/or available for children of certain age ranges, only the information for those households with observations for each pairwise combination of indicators is used.

**Table 2:** Number (and percentage) of households with no gender bias or with a bias for either boys or for girls in stunting and birth registration

*Source:* Author’s calculation based on MICS data.

*Note:* Percentages are expressed as a share of the total number of households

## 4 Results

This section presents the results by indicator and looks at patterns in the findings across countries. Given that the sample of countries and indicators relies on data availability, these results cannot be considered to be representative of the developing world or any country sub-grouping. The group averages presented in the results should be treated as such, recalling that the range of results can vary considerably. Of the twenty countries, seven are Low-Income (LIC),^15^ eight are Lower Middle Income (LMIC), four Upper-Middle Income (UMIC) and one had reached High-Income (HIC) status (Trinidad and Tobago). Their populations range from 329 thousand people in Belize to over 163 million people in Nigeria—the most populous country in the sample. Their demographic structure also varies, with Burundi having the largest share of children (about half of the population is 15 years old or younger), and Bosnia and Herzegovina at the other extreme, with only 16% of people below the age of 16.

Comparisons across countries are not straightforward for another two reasons: firstly, as noted, differences in average household sizes in particular may affect the assessment of inequality. Secondly, the definition and measurement of indicators, although mostly standardized by UNICEF, are not always kept, especially in earlier rounds of the surveys, leading to differences in the way the information is captured for some countries. The results for individual countries can be found in tables in Appendix 3. Summary statistics can be found in Appendix 5. This section concludes by analyzing the degree to which gender biases are jointly distributed within households.

Total inequalities between girls and boys across indicators of child wellbeing are of varying magnitude. On average, across all countries and years the Gini coefficient for working hours is 0.85, showing a large degree of inequality. Inequality in stunting is similarly high: on average, the Gini coefficient is 0.78 for this indicator. The Gini coefficient for birth registration and for school attendance is 0.47. Intrahousehold inequalities are also quite different across indicators and countries. Sections 4(a)–(d) examine how much of this overall inequality can be explained by differences within households.

### (a) Nutrition (stunting)

A strong body of evidence shows the detrimental effects of undernutrition. It is a risk factor for poor motor and cognitive child development (Black *et al.*, 2013), which in turn lowers educational attainment and carries into adulthood, directly affecting labor productivity and life-long earnings. The harmful effects of malnutrition also carry over from mothers to children and compromise maternal health (Bhalotra & Rawlings, 2011). Although different indicators can be used to determine whether a child is malnourished. Stunting (height-for-age) reflects better the cumulative effects of nutrition deprivation and thus is a better indicator of chronic malnutrition (Black *et al.*, 2013).^16^

On average, for all 15 countries and periods in the sample, 24% of boys and 23% of girls are stunted (summary statistics for all indicators are available in Appendix 5), figures that are consistent with previous evidence showing that differences in nutrition between girls and boys are not generally very large (UNICEF 2011). At the country level, stunting rates for boys range from 5% (Serbia, 2010) to 41% (Lao and Albania, 2000), and for girls, from 3% (Serbia, 2010) to 46% (Albania, 2000).

Even if, on aggregate, girls are as likely to be undernourished as boys, this could still hide other inequalities. Controlling for the age of children in the households, and looking at the ratio of stunting prevalence of girls to boys within households, the analysis here shows that on average for all countries about 78% of households have a bias for boys and 21% a bias for girls. Less than 1% of households have no bias in favor of children of either gender (see Appendix 4 for all countries and indicators). The percentages of households with and without biases differ, but the pattern of male bias is similar across countries. Moreover, these differences are large, so this results in a significant difference between the shares of households favoring boys and girls (see also Figure 2).

**Figure 1:**
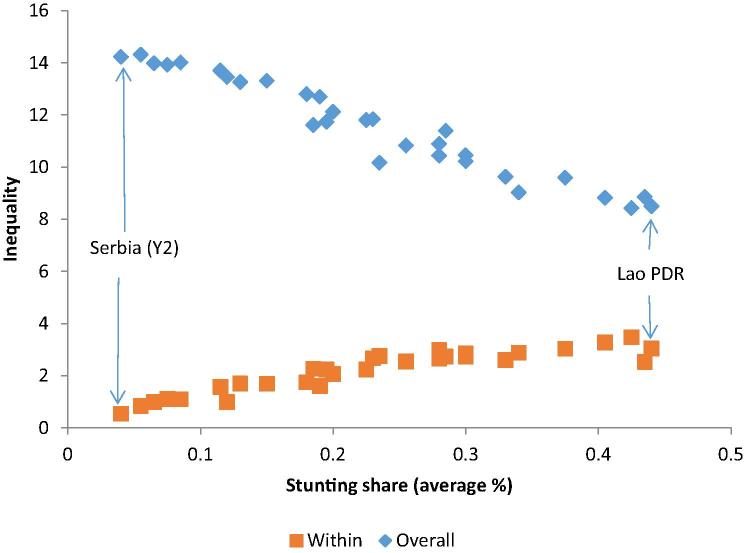
Average levels and inequality in stunting.

*Source:* Author’s calculation based on Multiple Indicator Cluster Survey (MICS) data. *Note:* Stunting average is the mean stunting levels for all children in the sample. Overall and within household inequalities refer to the L-Theil index results in each country.

Intrahousehold inequality varies across average levels of wellbeing and in relation to total inequality (Figure 1). Pooling all country-year observations, the Figure shows that where average stunting levels are higher, total inequality is lower. However, the opposite occurs with within-household inequality, which is higher where average stunting is higher in absolute and relative terms. For instance, in Lao People’s Democratic Republic (Lao PDR), a country with high levels of stunting, close to 40% of inequality occurs within households. The opposite occurs in countries like Serbia. This suggests that for nutritional outcomes, intrahousehold inequality should be a stronger concern in countries with higher levels of deprivation.

According to the inequality decomposition of the Theil index, on average 80% of the inequality in stunting rates can be attributed to inequality across households, whereas 20% occurs within households. However, in seven countries (Nigeria, Albania, Togo, Lao PDR, Sierra Leone, Swaziland, and Gambia) in both periods, the within-household component contributes to more than 20% of the total inequality, reaching 41% in Lao PDR.

**Figure 2:**
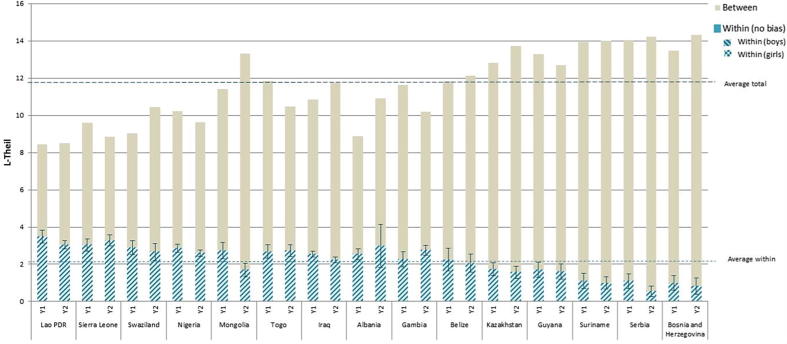
Inequality decomposition of stunting.

*Source:* Author’s calculation based on MICS data.

In six of the 15 countries with stunting data, overall inequality measured by the Theil index increases between the two periods; in four countries, it decreases; and in five countries, it remains virtually unchanged. But overall, as seen in Figure 2, there is little change in stunting inequality and its relative components from the first to the second period. Within-household inequality falls only in two countries (Mongolia and Iraq). Yet, neither of them managed to reduce total inequality because of a rise in between-household differences, and total inequality remained high (the Gini coefficient for Mongolia rose from 0.72 to 0.85 while in Iraq it remained virtually at the same level). For the rest of the countries, the change in the within-household component of inequality is not statistically significant and thus changes in total inequality are driven by the between-household component.

### (b) Birth registration

Unregistered children are deprived of their right to have an identity and may not be able to claim services and protections on an equal basis with other children. Birth registration is costly and difficult for some families. In some countries, parents need to pay a fee to register their children; in others, late registration carries a sanction that can place a heavy economic burden on the family, or may involve other external costs incurred through travel or accommodation and loss of earnings and work time. Sometimes the barriers are not monetary. For example, in Bhutan, children whose father is unknown cannot be registered, and in Indonesia, a marriage certificate is required to register a child’s birth (UNICEF, 2014). It is possible that given these difficulties, parents may not always be willing or able to register all their children. They may choose to register only one child, who may be either randomly selected by chance or circumstances or more instrumentally chosen to allow them access to services which could help them to support their family in the future.

On average, for the 19 countries analyzed, 53% of girls and 54% of boys are registered, but with large differences across countries, ranging from 2% in Trinidad and Tobago (2006) to 90% in Guyana (2006–07). On average, the percentage of children registered increases for girls and boys alike, from 50% in the first year in which registration was measured, to about 57% in the second. Again the actual rates differ in each country, but the similar trend for boys and girls is common. Disparities inside the household in terms of ratios of registration for girls and boys occur in about 98% of households, and in most countries there is a bias favoring girls (about 65% of cases on average). Only in Iraq does the bias run in the opposite direction, with less than a quarter of households favoring girls.

**Figure 3:**
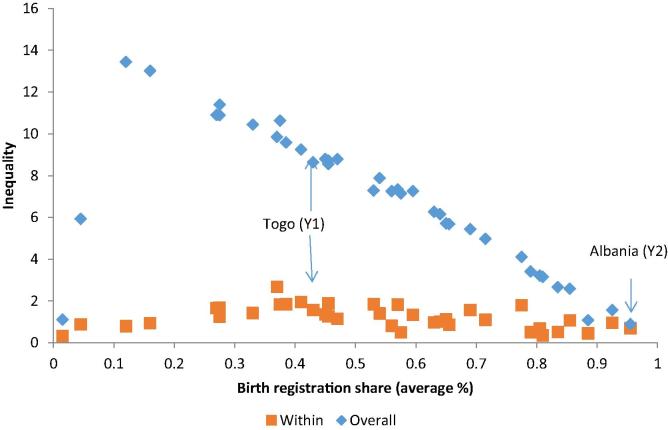
Average levels and inequality in birth registration.

*Source:* Author’s calculation based on MICS data. *Note:* Average levels of birth registration are the mean birth registration levels for all children in the sample. Overall and within household inequalities refer to the L-Theil index results in each country.

Figure 3 shows that the higher the average birth registration in the country, the lower the total inequality in absolute terms (e.g., Albania). The relationship with within-household inequality is less clear; if anything, within-household inequality is also slightly higher for countries in the middle of the distribution (e.g., Togo).

The between-household component accounts for 78% of total inequality, whereas the remaining 22% corresponds to inequality within households. There is a 20% or higher share of within-household inequality in both periods in Togo, Iraq, Mongolia, and Guyana. For eight countries, the share is below 20% in both periods while for the remaining seven countries, it fluctuates above and below 20% across the two time periods.

**Figure 4:**
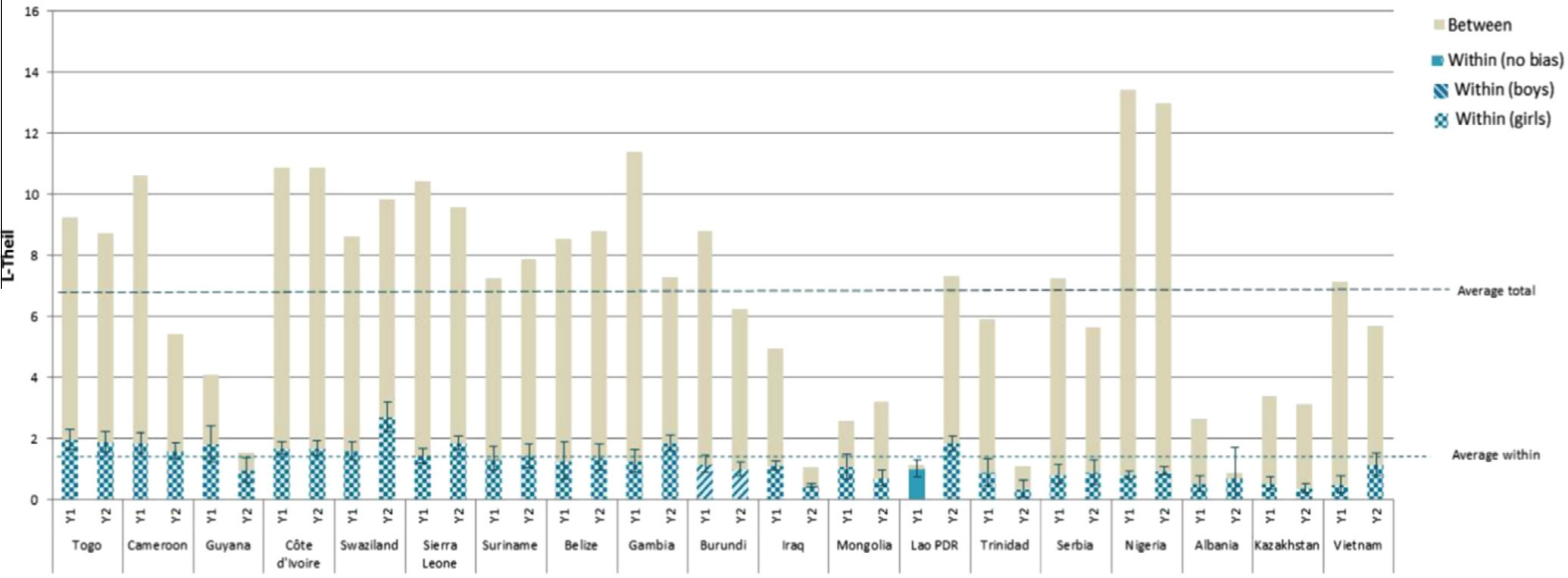
Inequality decomposition of birth registration.

*Source:* Author’s calculation based on MICS data.

With the general increase in birth registration rates, overall inequality falls over the two periods. Of the 19 countries with birth registration data, overall inequality between the two periods decreases in eight countries but increases in two (Lao PDR and Swaziland) (Figure 4). In the remaining nine countries, inequality remains virtually unchanged. The within-group component rises sharply, from 17% of total inequality in the first period to 25% in the second. The share of within-household inequality increases to above 20% in seven countries, although this change is only statistically significant in Vietnam, Lao PDR and Swaziland. Total inequality accompanies that upward trend in Lao PDR and Swaziland. Only in Iraq, within-household inequality decreases between the two periods; that reduction is accompanied by a reduction in total inequality.

### (c) School attendance

Education is critical to strengthening people’s capabilities and freedoms (Sen, 2001). Education can be a route out of poverty; an extra year of schooling can increase a person’s earnings, lead to better employment, reduce the chances of falling back into poverty, and is also linked to better health (Hannum and Buchmann, 2005, Harper et al., 2003).

The school attendance indicator refers to the number of children reported going to school (primary, or secondary) during the year of the survey. It is a gross attendance rate, because it includes all children regardless of whether they are attending the appropriate level of education for their age. It does not control for attrition levels or the quality of education, which can vary substantially. Further indicators would be needed to incorporate these important aspects of children’s right to education, where starker inequalities could be present.

Several factors can restrict access to education for some children. These range from affordability and physical access barriers (i.e., distance to schools) to social and cultural barriers, which can play differently for boys and girls. Reducing distance to school, for example, had a significant effect in increasing girls’ attendance in secondary schools in rural Tanzania, although it had less of an impact for boys (Burke & Beegle, 2004). Similarly, in terms of school attendance, disabilities tend to be less important for boys than for girls—a result of the way they interact with perceptions about gender roles and the lower value that parents place on their girls’ education (Rousso, 2003).

**Figure 6:**
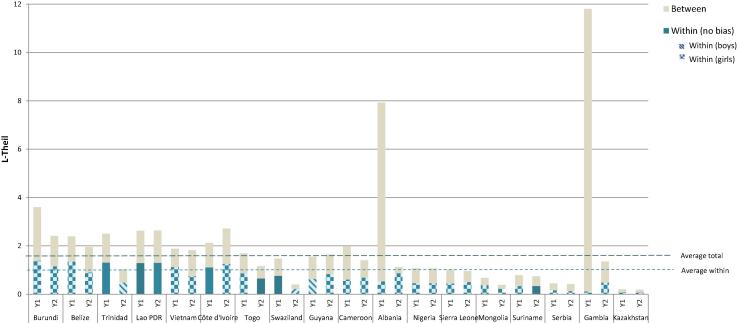
Inequality decomposition of school attendance.

*Source:* Author’s calculation based on MICS data.

On average, school attendance exceeds 80% for both boys and girls for the 18 countries with data, but again the range is wide across the sample. For boys, the range is from 44% in Gambia to 96% in Cameroon, while for girls the range is from 45% to 94% for the same two countries, respectively. In half the number of countries school attendance rates increase between the two periods for girls and boys alike. Once controlling for the different age composition of households, most households have some bias in the distribution of schooling and, interestingly, it runs in favor of girls in both periods for 11 of the 18 countries (see Figure 6). However, the differences are not as pronounced as they are for the remaining indicators, on average 53% of households favor girls and 46% favor boys.

**Figure 5:**
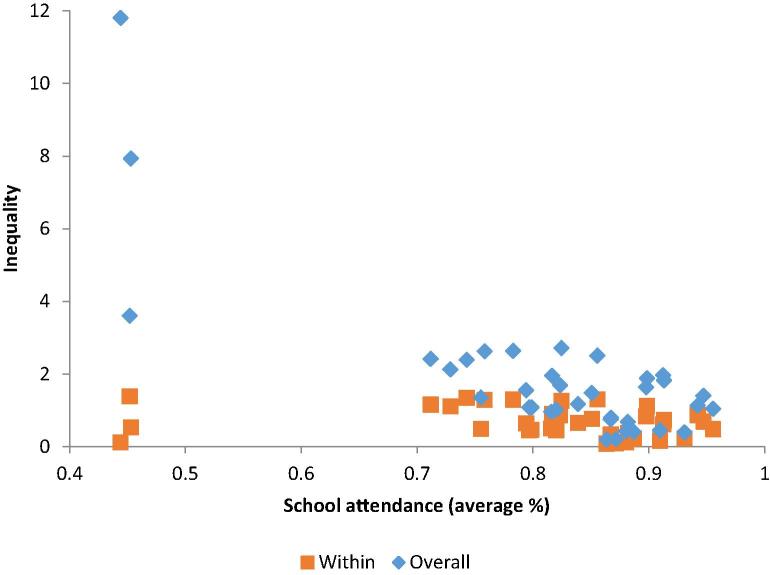
Average levels and inequality in school attendance.

*Source:* Author’s calculation based on MICS data. *Note:* Average school attendance is the mean school attendance levels for all children in the sample. Overall and within household inequalities refer to the L-Theil index results in each country.

There is no clear pattern between average progress in school attendance and either total or within-household inequality (Figure 5), in contrast to the other indicators of child wellbeing. The absolute levels of total and within household inequality are roughly similar in countries with lower and higher average rates of school attendance. However, when deprivations are low, intrahousehold inequality accounts for a greater share of total inequality, even if its absolute magnitude is smaller. This suggests that even if average deprivation is low, within-household inequality can be the main barrier to closing the gap and ensuring schooling for all children.

In fact, for this indicator, the share of within-household inequality is 45%, meaning that the within-household component accounts for almost half of the total inequality. For three countries (Togo, Mongolia and Swaziland), the within-household component makes the largest contribution to inequality in both periods.

On average, total inequality falls over the two periods. In 8 of the 18 countries with schooling data, overall inequality decreases (Figure 6).^17^ In one country (Côte d’Ivoire) inequality rises between the two periods of time, while for the remaining countries the change is not statistically significant. The distribution of inequality also changes with the general increases in school attendance across the countries. Within-household inequality falls in Trinidad and Tobago and Swaziland, which also show reduced overall inequality, but significantly increases in one country (Gambia), where the within-household inequality jumps from less to one to 36% mainly because the large drop of the between-household component. The changes in within-household inequality are statistically insignificant in the remaining 15 countries.

### (d) Working hours (economic, domestic, and chores)

Many children engage in work activities. Some work to ‘help their families in ways that are neither harmful nor exploitative, but others are put to work in ways that interfere with their education, drain their childhood of joy and crush their right to normal physical and mental development’ (UNICEF, 2014). Most children work and perform domestic duties for their parents or their own households (Edmonds & Pavcnik, 2005). Education and leisure form part of children’s fundamental rights: regardless of whether or not the activity produces economic value, both paid and unpaid work and household chores such as cooking, cleaning, or caring for other children are a drain on the time children have to learn and play. The term ‘work’ is used hereafter to refer to the sum of the time spent doing economic work, domestic work, and chores.

Child labor is typically measured with reference to a threshold of hours a child is engaged in economic activity; the thresholds to classify work as child labor vary with children’s age. However, such cut-offs can be arbitrary. They carry assumptions about an ideal minimum age of work as well as the amount of time children should have free for education and leisure. For this reason, this study does not use this definition of child labor, preferring instead to measure it by the total number of hours that children spend on these activities. Arguably, this still does not capture the extent to which child work is harmful for child wellbeing (Edmonds & Pavcnik, 2005), for example, it does not measure the degree to which children stop attending school because of work, but this is difficult to measure with multi-topic surveys such as MICS.

On average, across all 12 countries girls spend more hours a week (12 h) working and doing chores compared to boys (10 h),^18^ but this includes countries like Suriname, where boys and girls alike work only 0.31 h a week, and Cameroon, where boys spend more than 26 h and girls more than 31 h each week working. In seven of the 12 countries, the time that children (both girls and boys) spend working reduces between the two periods. In Nigeria, the reduction is only significant for boys, while in Gambia, there is an increase in the average number of hours that girls work (of more than three hours per week).

**Figure 8:**
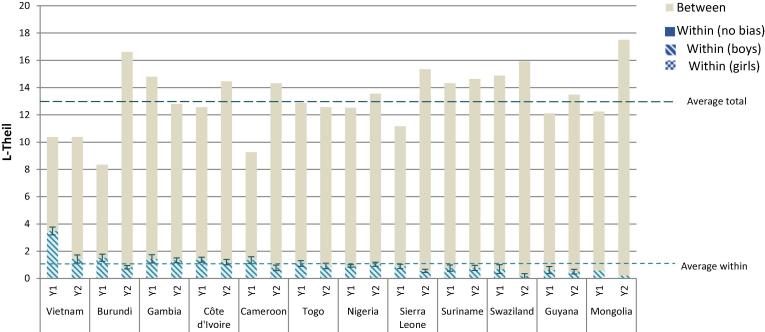
Inequality decomposition of working hours.

*Source:* Author’s calculation based on MICS data.

The longer hours worked by girls is also reflected when looking at the share of households who show a bias for boys in this indicator (see Figure 8). On average, across all countries and periods, girls spend less time working or doing chores in only 14% of the households, while boys spend less time in 86% of households.

**Figure 7:**
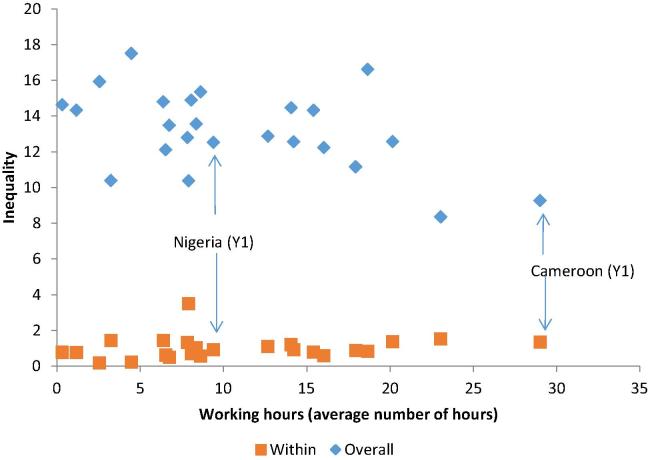
Average levels and inequality in working hours.

*Source:* Author’s calculation based on MICS data. *Note:* The average number of working hours is the mean hours worked by all children in the sample. Overall and within household inequalities refer to the L-Theil index results in each country.

Working hours follow a similar pattern to stunting. The higher the average number of hours worked by children, the lower the total inequality, but within-household inequality is of a fairly similar magnitude across countries (Figure 7). For example, while total inequality is lower in Cameroon than in Nigeria, intrahousehold inequality is of a similar absolute magnitude in both countries. In relative terms, the share of intrahousehold inequality seems to be large in countries where children work more hours.

Most inequality in working hours is accounted for by inequality across households; only 8% of inequality occurs within them. For this indicator, other group-based inequalities, such as location (urban/rural), economic conditions and regional differences may be more important in explaining inequalities. Only for Vietnam is this not the case: within household inequality is 34% in the first period and 14% in the second.

Although the number of working hours decreases over the two periods, inequality increases slightly. The Gini index on average is 0.81 in the first year and 0.89 in the second. In fact, total inequality increases in eight of the 12 countries and decreases only in one (Gambia). Within-household inequality on the other hand, falls in absolute and relative terms in six countries (Burundi, Cameroon, Mongolia, Sierra Leone, Swaziland, and Vietnam) and increases in none (Figure 8).^19^

### (e) Intrahousehold inequality as a barrier to ‘leaving no one behind’

Although the sample of countries and indicators is limited in many respects, the analysis of household survey data is illustrative of the presence of inequalities in four dimensions of child rights and wellbeing. Analyzing when such differences exist within households in the realization of children rights is an important aspect of identifying the barriers to ‘leaving no one behind’ and eliminating child poverty.

The analysis of the four variables of child wellbeing used here shows that small aggregate differences between girls and boys often obscure intrahousehold inequalities. In the aggregate, inequality is particularly high for stunting and working hours. The decomposition of the inequality index (L-Theil) shows that a large amount of inequality can occur within households, but with significant variation by country and indicator. In some areas, mainly work time, inequality occurs mostly between households. In contrast, inequalities inside households are particularly high for school attendance. They account for close to half of total inequality (over 40%) in 13 out 18 of countries, and in a further three countries, for more than half of total inequality in at least one of the periods. Within-household inequality in stunting and birth registration accounts for around one-fifth of total inequality on average for both periods.

The results show that intrahousehold inequality represents between six and 48% of total inequality across the four indicators when looking at averages across all countries. The variability by countries and years is high: the contribution of intrahousehold inequality is lowest in Gambia, Swaziland, and Mongolia (1% in the distribution of school in Gambia and of work time in Swaziland and Mongolia), and highest in Albania (79% in the distribution of birth registration).

Table 3 shows a summary of how the levels of total and within-household inequality evolve with higher levels of wellbeing. Only in countries with lower levels of stunting is there a lower level of within-household inequality. In the remaining three indicators that is not the case. In other words, there appears to be no automatic fall in absolute intrahousehold inequality in countries progressing toward higher child wellbeing Moreover, within-household inequalities can be increasingly important in relative terms, accounting for a greater share of total inequality in birth registration in particular, and in school attendance, to a lesser degree. For example, in schooling, where deprivations are relatively low, the residual gaps are mainly within households rather than across them, highlighting once again the relevance of addressing this type of inequality. This means it is not possible to eliminate child poverty and secure the rights of all children unless disparities within households are addressed. For stunting and working hours, within-household inequalities are less important in relative terms when deprivations are lower.

**Table 3:** Direction of inequality with higher levels of wellbeing

Indicator	Total inequality	Within-household inequality (absolute)	Share of within-household inequality (relative)
Stunting	↑	↓	↓
Birth registration	↓	↔	↑
School attendance	↔	↔	↑
Working hours	↑	↔	↓

*Note:* The arrows indicate the direction of the levels of inequality (↑ higher, ↓ lower or, ↔ stable) at higher levels of wellbeing.

### (f) Is there evidence of systematic bias against boys or girls?

Previous sections presented a detailed analysis of inequality for each indicator separately. It shows that for most countries, the bias in some indicators favors girls, and in others, it favors boys. Thus, at the country level, it is hard to conclude that there is a systematic bias against either gender. This section analyzes their joint distribution: in other words, it checks whether households tend to favor girls (or boys) in all areas of wellbeing, or rather to compensate for underinvestment in one area with overinvestment in another.

The four outcomes presented here are likely to be connected to each other. A birth certificate or identity card is often an administrative requirement for accessing essential services of health and education, so biases in this indicator are expected to be correlated with those in schooling and malnutrition. Similarly, the links between nutrition and education have been widely explored (Maluccio et al., 2009, Smith and Haddad, 2015). But outcomes in the different dimensions do not always go hand-in-hand. For example, Edmonds and Pavcnik (2005) find that child work is not necessarily incompatible with schooling, in fact, only for children working in excess of 40 h a week there was a significant drop in school attendance.

Table 4 shows the results of the exercise of assessing the degree of correlation of inequality in the four dimensions. In five of the six possible combinations of indicators, households show a preference for boys, and in one, they show a preference for girls. The average across indicators also shows that more households favor boys over girls in two indicators at the time. Starting with these, 79% of households that tend to favor boys over girls in terms of nutrition (stunting) also favor them in terms of birth registration. The respective percentages are 71% for stunting and school attendance; 88% for stunting and working hours; 47% for birth registration and school; 86% for birth registration and work; and 84% in school and work.

**Table 4:** Measures of association

Variables	*P* statistic for boys	*P* statistic for girls	Absolute difference
Stunting/birth registration	0.79	0.71	0.07
Stunting/school attendance	0.71	0.52	0.19
Stunting/working hours	0.88	0.33	0.55
Birth registration/school attendance	0.47	0.74	(0.27)
Birth registration/working hours	0.86	0.75	0.12
School attendance/working hours	0.84	0.58	0.26
Average	0.76	0.61	0.15

*Note:* Underlined values show whether the *P* statistic is higher for boys or girls. Where the absolute difference is in brackets, in indicates that the value for girls is higher than for boys.

*Source:* Author’s calculation based on MICS data.

On average, fewer households favor girls in two indicators at the time, but it is noticeable that in three of the cases where the *P* statistic indicates a preference for boys, the absolute difference between the *P* statistic for boys and for girls is relatively small (below 0.20). In some cases, the proportion of households favoring girls is considerable. It is 71% for stunting and birth registration; 52% for stunting and school attendance; 33% for stunting and work; 74% for birth registration and school; 75% in birth registration and work; and 58% in school and work.

These results vary across countries (Appendix 7), with some having a more distinctive pattern than others. For example, take the case of the positive bias for girls in stunting and birth registration. With the pool of observations from all countries, the *P* statistic is 0.71, but this ranges from 0.52 in Mongolia to 0.91 in Nigeria. Similarly, the bias for boys in school attendance and work time ranges from 0.76 in Vietnam to 0.96 in Swaziland.

In Lao PDR and Trinidad and Tobago, most pairings favor girls, while in the remaining 15 countries, most pairings favor boys.^20^ For these countries, the absolute difference between the *P* statistic for boys and for girls ranges from 0.24 in Kazakhstan to 0.02 in Belize, showing that in the first country there is stronger evidence for boy preference than in the latter. In Albania and Nigeria, the same number of pairings favors girls and boys, but on average, most households favor girls in Albania, whereas most favor boys in Nigeria.

As mentioned in the Introduction, previous studies have produced varying evidence on intrahousehold distributions and the directions of biases, and this study seems to confirm the evidence by systematically examining cross-country data. While there is some overall bias for boys, this is not universal across indicators nor countries. In particular, it is possible that some household characteristics are systematically associated with a more unequal distribution of resources between boys and girls. However, it is likely that these patterns vary across countries and indicators of child wellbeing. The variability in intrahousehold inequality across countries indicators found in this study suggests that biases may respond to different aspects in different countries and may relate to different social gender norms and household institutions. A more in-depth analysis would be needed to uncover the specific mechanisms that drive intrahousehold inequalities in each of the dimensions of child wellbeing presented in this study.

## 5 Discussion and conclusions

Progress in improving many child wellbeing dimensions has occurred across the globe (UNICEF, 2014). However, the way in which progress happens may not be equitable and the patterns of inequality vary across dimensions of wellbeing. This paper provides an innovative methodological approach to measuring the extent of intrahousehold inequalities, presenting a broader picture of child wellbeing and its distribution across a sample of 20 developing countries. In all indicators of child wellbeing there have been improvements, but the patterns of distribution that emerge from these improvements are very different. Overall, the paper advances five main findings.

First, assessing inequality, and in particular that which occurs within households, is important, even in the context of country progress toward the realization of child rights and wellbeing. When comparing averages between girls and boys, while small differences are noted in many areas of wellbeing, some important disparities remain. Across the sampled countries (12–19 depending on the indicator), the average Gini coefficient for school attendance and for birth registration is 0.47; it is 0.78 for stunting, and 0.85 for working hours. To close the gap between girls and boys, it is important to know where these disparities are located.

Second, by using a decomposable measure of inequality (the Theil index) it is shown that significant inequalities occur within households. Between-household inequality in working hours is relatively large (on average, 92% of total inequality) and thus addressing barriers to reduce inequality across households appear to be a priority for closing the gap in this indicator of child wellbeing. For the remaining variables, within-household inequalities are considerable. For stunting and birth registration they are close to a fifth of total inequality, and for school attendance, despite impressive progress, over 40% of gender inequalities occur within households.

Third, even when they are lower in absolute terms, intrahousehold inequalities might still be considered a priority in terms of an agenda focused on ‘leaving no one behind’. Although the relatively small timeframe (around five years) and country sample are perhaps insufficient to capture long-term global trends in inequality, looking at how inequality stands for countries at different levels of wellbeing can be illustrative of these trends. Where average levels of child wellbeing increase and total inequality falls, within-household inequalities are more important in relative terms, accounting for a larger share of the total inequality. For example, the analysis shows that intrahousehold inequality in birth registration and school attendance tends to be higher in countries where total inequality is lower, suggesting that the gaps that are more difficult to address may be located inside households. These results indicate that it is not possible to eliminate child poverty and secure the rights of all children unless disparities within households are addressed. A further avenue for research would examine these trends alongside other country characteristics that have been pointed in the literature as mechanisms for the perpetuation of gender biases inside households.

Fourth, evidence from a multiplicity of separate county studies had shown that the direction of the gender bias is not universal. Using data from a wider yet diverse set of developing counties the present study is useful to confirm that such conclusion holds when using a consistent methodology and dataset; disparities inside households do not follow the same bias toward one or the other gender and that the direction of the bias is not the same across indicators of wellbeing. For example, in stunting and work hours, most households have a bias for boys while in school attendance and birth registration, most households tend to favor girls. When looking at pairs of indicators, in five of the possible six combinations the majority of households show a preference for boys, and in only one there is an overall preference for girls. Even then, for some pairings, a considerable proportion of households show a preference for girls. The methodology presented in this paper is able to show correlations between dimensions of wellbeing, but is not sufficient to suggest a channel of causality.

Fifth, the gender bias is varied across countries, with some showing a more distinctive preference pattern than others. This has been found elsewhere and suggests that biases respond to different social norms and household institutions. Institutions and norms surrounding gender roles, patterns of inheritance, marriage, and divorce, all matter to understand the varying degree and direction of intrahousehold inequality bias. Yet these too are likely to differ across countries. Quantitative analysis to examine what drives intrahousehold inequality on a cross-country basis could contribute to future research. It may be necessary to complement this research with the already flourishing literature that explores on a country basis the social values and norms, as well as the economic logic, which underpin these inequality patterns.

This paper is an important contribution to measuring the extent of such inequalities using a systematic methodology and data for 20 developing countries. As pointed out 25 years ago, the neglect of intrahousehold inequality affects the assessment of the levels of poverty, and could lead to a skewed view of the patterns of progress (Haddad & Kanbur, 1990). As with any inequality analysis, a crucial question remains about whether the magnitudes found should be a cause of concern. This question is hard to answer when only one dimension of wellbeing is under consideration. The methodological approach proposed in this study, aimed to provide a broader picture of child wellbeing and its distribution can provide an avenue to address this question. When biases persist across wellbeing dimensions, the unfairness of inequalities and the imperative for action are stronger.

While the number of indicators available to carry out a multidimensional analysis is relatively limited compared to what the CRC defines as the dimensions of child wellbeing, it is a good starting point to go beyond the measurement of a single dimension or using a summary indicator of child wellbeing. The methodology presented in the paper could be applied to more complete national-level datasets in future applications. Furthermore, additional cross-country data which allows for this type of distributional analysis with a broader range of indicators would be a valuable contribution to future research. The release of UNICEF’s next round of MICS surveys could be the starting point for this.

The varying and sometimes large amount of intrahousehold inequality found in most countries poses difficulties for policy-making. Interventions to address inequalities in child wellbeing may need to be targeted more specifically at individuals or sub-groups within households rather than at households in general (Haddad and Kanbur, 1992, Roemling and Qaim, 2013, Sahn and Younger, 2009), and at breaking the most entrenched patterns of systematic disadvantages across various dimensions of child wellbeing.

## Figures and Tables

**Table:** 

Country	Year of fieldwork	Income level (WB classification)	Population (1,000)	% Below 16 years old^a^
Albania	2000/2005	LM/LM	3122/3082	32/28
Bosnia and Herzegovina	2006/2011	LM/UM	3839/3832	17/16
Belize	2006/2011	UM/LM	291/329	41/38
Burundi	2000/2005	L/L	6767/7934	51/48
Cameroon	2000/2006	L/LM	15,928/18,597	48/47
Côte d’Ivoire	2000/2006	L/L	16,518/18,486	46/46
Gambia	2000/2005–06	L/L	1229/1488	48/48
Guyana	2000/2006–07	LM/LM	742/746	38/39
Iraq	2006/2011	LM/LM	27,717/31,868	44/44
Kazakhstan	2006/2010–11	UM/UM	15,603/16,554	27 /26
Lao People’s Democratic Republic (PDR)	2000/2006	L/L	5343/5839	46/43
Mongolia	2000/2005	L/L	14,957/15,452	37/31
Nigeria	2007/2011	L/LM	147,153/163,771	46/46
Serbia	2005–06/2010	UM/UM	5243/5776	20 /19
Sierra Leone	2005/2010	L/L	9187/9059	46/46
Suriname	2006/2010	LM/UM	496/518	32/30
Swaziland	2000/2010	LM/LM	1064/1193	47/41
Togo	2000/2006	L/L	4875/5732	47/46
Trinidad and Tobago	2000/2006	UM/H	1268/1303	28/24
Vietnam	2006/2010–11	L/LM	84,980/89,322	29/26


**Table 5:** Absolute and relative within and overall inequality

**(a) Stunting**																				
	Kazakhstan			Nigeria			Albania			Bosnia and Herzegovina			Togo			Suriname			Belize	
Absolute	Y1	Y2		Y1	Y2		Y1	Y2^		Y1	Y2^		Y1	Y2		Y1	Y2		Y1	Y2
Within	1.75	1.57		2.85	2.60		2.53	2.98		0.99	0.84		2.67	2.74		1.11	1.00		2.25	2.06
Overall	12.80	13.70	⁎	10.22	9.63	⁎	8.85	10.89	⁎	13.45	14.32	⁎	11.84	10.46	⁎	13.93	13.98		11.81	12.12
Relative																				
Share of within (%)	0.14	0.11		0.28	0.27		0.29	0.27		0.07	0.06		0.23	0.26		0.08	0.07		0.19	0.17

	Iraq			Lao			Mongolia			Serbia			Sierra Leone			Swaziland			Guyana			Gambia		
Absolute	Y1	Y2		Y1	Y2		Y1^	Y2		Y1	Y2		Y1	Y2		Y1	Y2		Y1	Y2		Y1	Y2	
Within	2.54	2.23	⁎	3.48	3.04		2.73	1.70	⁎	1.10	0.54		3.03	3.28		2.88	2.66		1.70	1.61		2.27	2.75	
Overall	10.83	11.74	⁎	8.43	8.50		11.39	13.31	⁎	14.02	14.23		9.59	8.82	⁎	9.03	10.44	⁎	13.27	12.70		11.61	10.17	⁎
Relative																								
Share of within (%)	0.23	0.19		0.412	0.36		0.24	0.13		0.08	0.04		0.32	0.37		0.32	0.25		0.13	0.13		0.20	0.27	

Average	All	Y1	Y2
Absolute			
Within	2.18	2.26	2.11
Overall	11.54	11.40	11.67
Relative			
Share of within (%)	0.21	0.21	0.20
Share of between (%)	0.79	0.79	0.80

**(b) Birth registration**																				
	Kazakhstan			Nigeria			Albania			Burundi			Cameroon			Vietnam			Togo	
Absolute	Y1	Y2		Y1	Y2		Y1	Y2^		Y1	Y2		Y1	Y2		Y1	Y2		Y1	Y2
Within	0.51	0.36		0.79	0.94		0.51	0.70		1.15	0.98		1.84	1.57		0.49	1.14	⁎	1.95	1.88
Overall	3.41	3.15		13.44	13.02	⁎	2.66	0.89	⁎	8.80	6.27	⁎	10.63	5.44	⁎	7.15	5.71		9.24	8.72
Relative																				
Share of within (%)	0.15	0.11		0.06	0.07		0.19	0.79		0.13	0.16		0.17	0.29		0.07	0.20		0.21	0.22

	Côte d’Ivoire			Suriname			Belize			Trinidad and Tobago			Iraq			Lao			Mongolia		
Absolute	Y1	Y2		Y1	Y2		Y1	Y2		Y1	Y2		Y1	Y2		Y1	Y2		Y1^	Y2	
Within	1.66	1.68		1.34	1.41		1.27	1.36		0.88	0.33		1.11	0.45	⁎	1.01	1.83	⁎	1.07	0.69	
Overall	10.90	10.89		7.26	7.88		8.56	8.80		5.93	1.12	⁎	4.98	1.08	⁎	6.16	7.33	⁎	2.59	3.22	
Relative																					
Share of within (%)	0.15	0.15		0.19	0.18		0.15	0.15		0.15	0.30		0.22	0.42		0.16	0.25		0.41	0.22	

	Serbia			Sierra Leone			Swaziland			Guyana			Gambia		
Absolute	Y1	Y2		Y1	Y2		Y1	Y2		Y1	Y2		Y1	Y2	
Within	0.81	0.87		1.43	1.85		1.57	2.68	⁎	1.80	0.95		1.25	1.85	
Overall	7.26	5.68		10.44	9.59		8.64	9.85	⁎	4.11	1.56	⁎	11.40	7.29	⁎
Relative															
Share of within (%)	0.11	0.15		0.14	0.19		0.18	0.27		0.44	0.61		0.11	0.25	

Average	All	Y1	Y2
Absolute			
Within	1.21	1.18	1.24
Overall	6.87	7.56	6.18
Relative			
Share of within (%)	0.22	0.18	0.26
Share of between (%)	0.78	0.82	0.74

**(c) School attendance**																									
	Kazakhstan		Nigeria			Albania			Burundi			Cameroon			Vietnam			Togo			Côte d’Ivoire			Suriname	
Absolute	Y1	Y2	Y1	Y2		Y1	Y2		Y1	Y2		Y1	Y2		Y1	Y2		Y1	Y2		Y1	Y2		Y1	Y2
Within	0.09	0.08	0.45	0.46		0.53	0.87		1.38	1.16		0.61	0.69		1.13	0.74		0.86	0.65		1.11	1.26		0.34	0.34
Overall	0.21	0.19	1.08	1.08		7.93	1.13	⁎	3.60	2.41	⁎	1.97	1.40	⁎	1.88	1.83		1.69	1.17	⁎	2.12	2.72	⁎	0.79	0.75
Relative																									
Share of within (%)	0.41	0.41	0.42	0.42		0.07	0.77		0.38	0.48		0.31	0.49		0.60	0.41		0.51	0.56		0.52	0.46		0.43	0.46

	Belize			Trinidad and Tobago			Lao			Mongolia			Serbia			Sierra Leone			Swaziland			Guyana			Gambia		
Absolute	Y1	Y2		Y1	Y2		Y1	Y2		Y1	Y2		Y1	Y2		Y1	Y2		Y1	Y2		Y1	Y2		Y1	Y2	
Within	1.34	0.90		1.30	0.48	⁎	1.28	1.29		0.38	0.23		0.16	0.13		0.44	0.50		0.77	0.22	⁎	0.64	0.83		0.11	0.49	⁎
Overall	2.39	1.95		2.51	1.04	⁎	2.62	2.64		0.68	0.39	⁎	0.45	0.42		1.02	0.96		1.47	0.40	⁎	1.55	1.64		11.81	1.35	⁎
Relative																											
Share of within (%)	0.56	0.46		0.52	0.46		0.49	0.49		0.56	0.58		0.37	0.30		0.44	0.52		0.52	0.55		0.41	0.51		0.01	0.36	

Average	All	Y1	Y2
Absolute			
Within	0.67	0.72	0.63
Overall	1.92	2.54	1.30
Relative			
Share of within (%)	0.45	0.42	0.48
Share of between (%)	0.55	0.58	0.52

**(d) Economic or domestic working hours**																	
	Nigeria			Burundi			Cameroon			Togo			Côte d’Ivoire			Suriname	
Absolute	Y1	Y2		Y1	Y2		Y1	Y2		Y1	Y2		Y1	Y2		Y1^	Y2^
Within	0.92	1.04		1.52	0.82	⁎	1.35	0.78	⁎	1.10	0.92		1.38	1.20		0.76	0.77
Overall	12.52	13.56	⁎	8.35	16.62	⁎	9.27	14.33	⁎	12.87	12.56		12.57	14.47	⁎	14.33	14.64
Relative																	
Share of within (%)	0.07	0.08		0.18	0.05		0.15	0.05		0.09	0.07		0.11	0.08		0.05	0.05

	Mongolia			Sierra Leone			Swaziland			Guyana			Vietnam			Gambia		
Absolute	Y1^	Y2^		Y1	Y2		Y1^	Y2^		Y1	Y2		Y1	Y2		Y1	Y2	
Within	0.58	0.22	⁎	0.88	0.56	⁎	0.69	0.19	⁎	0.62	0.50		3.49	1.43	⁎	1.44	1.33	
Overall	12.24	17.51	⁎	11.16	15.35	⁎	14.89	15.93	⁎	12.12	13.49	⁎	10.38	10.39		14.80	12.80	⁎
Relative																		
Share of within (%)	0.05	0.01		0.08	0.04		0.05	0.01		0.05	0.04		0.34	0.14		0.10	0.10	

Average	All	Y1	Y2
Absolute			
Within	1.02	1.23	0.81
Overall	13.17	12.13	14.22
Relative			
Share of Within (%)	0.08	0.11	0.06
Share of Between (%)	0.92	0.89	0.94

Source: Author’s calculation based on MICS data.

⁎ Indicates that the change from the first to the second year is statistically significant (5%).

^ Indicates that less than 10% of the original survey observations were kept for the analysis.

**Table 6:** Share of households by favored gender

**(a) Stunting (% of households)**																
	Kazakhstan		Albania		Belize		Bosnia and Herzegovina		Gambia		Guyana		Lao PDR		Mongolia	
	Y1	Y2	Y1	Y2	Y1	Y2	Y1	Y2	Y1	Y2	Y1	Y2	Y1	Y2	Y1	Y2
None	0.0	0.0	2.1	1.3	0.0	0.8	0.3	0.5	0.7	0.3	0.0	0.5	0.3	0.3	0.9	0.8
Boys	83.8	90.6	74.3	77.2	81.1	83.0	88.9	93.8	76.5	70.6	84.6	78.7	67.2	64.2	75.2	85.4
Girls	16.2	9.4	23.6	21.5	18.9	16.3	10.8	5.7	22.8	29.2	15.4	20.8	32.5	35.5	23.9	13.8
Probability > *F*	0.000	0.000	0.000	0.000	0.000	0.000	0.000	0.000	0.000	0.000	0.000	0.000	0.000	0.000	0.000	0.000

	Nigeria		Serbia		Sierra Leone		Suriname		Swaziland		Togo		Iraq	
	Y1	Y2	Y1	Y2	Y1	Y2	Y1	Y2	Y1	Y2	Y1	Y2	Y1	Y2
None	1.0	0.8	0.2	0.6	2.3	1.8	0.0	0.2	0.4	0.7	0.92	0.91	0.5	0.2
Boys	71.9	67.3	87.6	94.5	67.9	63.3	91.0	88.8	64.9	72.6	75.71	68.18	76.1	79.5
Girls	27.0	32.0	12.2	4.9	29.8	34.9	9.0	11.0	34.7	26.7	23.37	30.91	23.5	20.4
Probability > *F*	0.000	0.000	0.000	0.000	0.000	0.000	0.000	0.000	0.000	0.000	0.000	0.000	0.000	0.000

Average	All	Y1	Y2
None	0.6	0.6	0.6
Boys	78.1	77.8	78.5
Girls	21.2	21.6	20.9

**(b) Birth registration (% of households)**												
	Kazakhstan		Albania		Belize		Burundi		Cameroon		Côte d’Ivoire	
	Y1	Y2	Y1	Y2	Y1	Y2	Y1	Y2	Y1	Y2	Y1	Y2
None	2.2	1.9	4.2	3.8	0.0	0.4	0.8	1.0	1.0	2.2	0.7	1.0
Boys	39.2	40.7	27.8	34.8	24.2	21.1	26.6	33.1	21.1	36.6	21.1	21.3
Girls	58.7	57.4	68.1	61.4	75.8	78.5	72.7	66.0	77.9	61.2	78.3	77.8
Probability > *F*	0.000	0.000	0.000	0.001	0.000	0.000	0.000	0.000	0.000	0.000	0.000	0.000

	Gambia		Guyana		Lao PDR		Mongolia		Nigeria		Serbia	
	Y1	Y2	Y1	Y2	Y1	Y2	Y1	Y2	Y1	Y2	Y1	Y2
None	1.4	1.5	1.3	2.0	0.6	1.0	3.7	3.5	0.4	0.3	2.0	5.2
Boys	15.9	31.2	35.3	43.6	32.9	32.8	44.7	41.1	9.9	10.9	21.8	36.3
Girls	82.6	67.3	63.4	54.4	66.5	66.3	51.6	55.4	89.8	88.8	76.2	58.5
Probability > *F*	0.000	0.000	0.000	0.003	0.000	0.000	0.095	0.000	0.000	0.000	0.000	0.000

	Sierra Leone		Suriname		Swaziland		Togo		Trinidad and Tobago		Vietnam		Iraq	
	Y1	Y2	Y1	Y2	Y1	Y2	Y1	Y2	Y1	Y2	Y1	Y2	Y1	Y2
None	2.4	2.2	1.5	1.1	1.1	0.7	2.48	1.55	3.0	6.3	0.9	1.5	0.5	0.2
Boys	20.2	26.3	29.8	31.6	30.0	27.6	30.39	26.92	32.6	41.3	38.2	32.9	76.1	79.5
Girls	77.4	71.5	68.7	67.2	68.9	71.7	67.13	71.53	64.4	52.4	61.0	65.6	23.5	20.4
Probability > *F*	0.000	0.000	0.000	0.000	0.000	0.000	0.000	0.000	0.000	0.042	0.000	0.000	0.000	0.000

Average	All	Y1	Y2
None	1.8	1.6	2.0
Boys	32.3	30.4	34.2
Girls	65.9	68.0	63.9

**(c) School attendance (% of households)**												
	Kazakhstan		Albania		Belize		Burundi		Cameroon		Côte d’Ivoire	
	Y1	Y2	Y1	Y2	Y1	Y2	Y1	Y2	Y1	Y2	Y1	Y2
None	0.9	0.7	0.4	1.5	1.3	0.4	0.9	0.4	0.7	1.0	1.2	1.5
Boys	54.0	52.7	20.0	44.2	49.0	50.5	43.7	48.8	41.3	47.8	48.8	48.1
Girls	45.1	46.6	79.7	54.4	49.7	49.0	55.5	50.8	58.0	51.2	50.0	50.4
Probability > *F*	0.000	0.000	0.000	0.000	0.735	0.320	0.000	0.022	0.000	0.000	0.092	0.001

	Gambia		Guyana		Lao PDR		Mongolia		Nigeria		Serbia	
	Y1	Y2	Y1	Y2	Y1	Y2	Y1	Y2	Y1	Y2	Y1	Y2
None	0.58	1.18	1.3	0.6	0.3	0.5	0.6	1.3	0.6	1.0	1.5	3.5
Boys	8.61	47.12	44.5	49.3	50.2	50.3	45.1	45.4	46.9	46.2	41.8	44.4
Girls	90.81	51.7	54.3	50.1	49.6	49.2	54.3	53.4	52.4	52.7	56.7	52.1
Probability > *F*	0.000	0.000	0.000	0.523	0.493	0.044	0.000	0.000	0.000	0.000	0.000	0.001

	Sierra Leone		Suriname		Swaziland		Togo		Trinidad and Tobago		Vietnam	
	Y1	Y2	Y1	Y2	Y1	Y2	Y1	Y2	Y1	Y2	Y1	Y2
None	1.2	1.2	0.8	0.9	1.3	1.7	1.2	1.3	3.5	4.0	4.2	0.9
Boys	52.2	48.7	45.2	47.3	44.2	49.7	50.0	52.8	43.8	46.4	45.5	44.6
Girls	46.6	50.1	54.0	51.8	54.5	48.7	48.8	45.9	52.7	49.7	50.3	54.5
Probability > *F*	0.000	0.029	0.000	0.000	0.000	0.403	0.199	0.000	0.000	0.001	0.000	0.000

Average	All	Y1	Y2
None	1.3	1.2	1.3
Boys	45.5	43.0	48.0
Girls	53.2	55.7	50.7

**(d) Working hours (% of households)**												
	Nigeria		Burundi		Cameroon		Togo		Côte d’Ivoire		Suriname	
	Y1	Y2	Y1	Y2	Y1	Y1	Y1	Y2	Y1	Y2	Y1	Y2
None	0.3	0.2	0.6	0.0	0.4	0.4	0.4	0.0	0.2	0.0	0.7	0.6
Boys	83.4	87.2	75.0	93.0	85.1	85.1	89.5	97.6	88.3	91.4	87.5	94.1
Girls	16.3	12.7	24.4	7.0	14.6	14.6	10.0	2.4	11.6	8.6	11.9	5.3
Probability > *F*	0.000	0.000	0.000	0.000	0.000	0.000	0.000	0.000	0.000	0.000	0.000	0.000

	Mongolia		Sierra Leone		Swaziland		Guyana		Vietnam		Gambia	
	Y1	Y1	Y1	Y2	Y2	Y2	Y1	Y2	Y1	Y2	Y1	Y2
None	0.4	0.4	1.0	0.2	0.1	0.0	1.4	1.9	0.05	0.55	2.18	0.60
Boys	82.2	82.2	77.0	90.7	88.4	84.8	75.8	80.6	84.33	89.85	72.48	85.12
Girls	17.4	17.4	22.0	9.1	11.5	15.2	22.8	17.5	15.62	9.60	25.34	14.29
Probability > *F*	0.000	0.000	0.000	0.000	0.000	0.000	0.000	0.000	0.000	0.000	0.000	0.000

Average	All	Y1	Y2
None	0.5	0.6	0.4
Boys	86.0	81.8	90.2
Girls	13.5	17.6	9.4

*Source:* Author’s calculation based on Multiple Indicator Cluster Survey (MICS) data.

**Table 7:** Average by indicators

**(a) Stunting (average %)**																								
	Kazakhstan			Nigeria			Albania			Bosnia and Herzegovina			Togo			Suriname			Belize			Iraq		
	Y1	Y2	∗∗	Y1	Y2		Y1	Y2		Y1	Y2		Y1	Y2		Y1	Y2		Y1	Y2		Y1	Y2	
Boys	0.19	0.10	⁎	0.31	0.33		0.41	0.30		0.12	0.06		0.24	0.32	⁎	0.08	0.07		0.23	0.17		0.26	0.20	⁎
Girls	0.17	0.13		0.29	0.33		0.46	0.26	⁎	0.12	0.05		0.22	0.28		0.07	0.06		0.22	0.23		0.25	0.19	⁎
++																								
*N*	1,006	1,248		5,357	10,247		288	158		714	403		1,301	1,873		612	1,053		254	522		6,979	16,968	

	Lao			Mongolia			Serbia		Sierra Leone		Swaziland		Guyana		Gambia	
	Y1	Y2	∗∗	Y1	Y2		Y1	Y2	Y1	Y2	Y1	Y2	Y1	Y2	Y1	Y2
Boys	0.41	0.44		0.28	0.14	⁎	0.09	0.05	0.38	0.43	0.38	0.29	0.13	0.19	0.21	0.25
Girls	0.44	0.44		0.29	0.16	⁎	0.08	0.03	0.37	0.38	0.30	0.27	0.13	0.19	0.16	0.22
++											+					
*N*	1,250	3,113		568	754		1,053	6,93	1,637	2,441	1,435	853	878	790	1,546	3,259

Average	All	Y1	Y2
Boys	0.24	0.25	0.22
Girls	0.23	0.24	0.21

**(b) Birth registration (average %)**																			
	Kazakhstan			Nigeria			Albania			Burundi			Cameroon			Vietnam		Togo	
	Y1	Y2	∗∗	Y1	Y2		Y1	Y2		Y1	Y2		Y1	Y2		Y1	Y2	Y1	Y2
Boys	0.79	0.82		0.13	0.16		0.83	0.92		0.48	0.63	⁎	0.38	0.69	⁎	0.56	0.63	0.42	0.44
Girls	0.79	0.80		0.11	0.16	⁎	0.84	0.99	⁎	0.46	0.63	⁎	0.37	0.69	⁎	0.59	0.67	0.40	0.47
++																			
*N*	1,006	1,248		5,257	10,224		288	158		1,017	2,193		1,463	2,545		7,06	547	1,294	1,866

	Côte d’Ivoire		Suriname		Belize		Trinidad and Tobago		Iraq			Lao	
	Y1	Y2	Y1	Y2	Y1	Y2	Y1	Y2	Y1	Y2		Y1	Y2
Boys	0.27	0.27	0.59	0.57	0.44	0.44	0.04	0.02	0.72	0.89	⁎	0.64	0.57
Girls	0.27	0.28	0.60	0.51	0.47	0.46	0.05	0.01	0.71	0.88	⁎	0.64	0.57
++													
*N*	3,188	3,535	608	1,053	252	522			6,979	16,968		1,257	3,105

	Mongolia		Serbia		Sierra Leone		Swaziland		Guyana			Gambia		
	Y1	Y2	Y1	Y2	Y1	Y2	Y1	Y2	Y1	Y2		Y1	Y2	
Boys	0.85	0.80	0.55	0.64	0.33	0.40	0.44	0.38	0.76	0.90	⁎	0.28	0.53	⁎
Girls	0.86	0.81	0.57	0.67	0.33	0.37	0.42	0.36	0.79	0.95	⁎	0.27	0.53	⁎
++														
*N*	568	754	1,051	691	1,625	2,439	1,415	842	880	788		1,536	3,251	

Average	All	Y1	Y2
Boys	0.53	0.50	0.56
Girls	0.54	0.50	0.57

**(c) School attendance (average %)**																
	Kazakhstan			Nigeria		Albania			Burundi			Cameroon			Vietnam	
	Y1	Y2	∗∗	Y1	Y2	Y1	Y2		Y1	Y2		Y1	Y2		Y1	Y2
Boys	0.87	0.85		0.80	0.80	0.46	0.96	⁎	0.47	0.73	⁎	0.91	0.96	⁎	0.90	0.91
Girls	0.87	0.87		0.79	0.80	0.45	0.92	⁎	0.43	0.70	⁎	0.91	0.94		0.90	0.92
++																
*N*	5,814	5,594		29,016	42,679	2,598	1,656		6,417	12,066		4,903	11,089		8,097	4,429

	Togo		Côte d’Ivoire			Suriname		Belize			Trinidad and Tobago			Lao		
	Y1	Y2	Y1	Y2		Y1	Y2	Y1	Y2		Y1	Y2		Y1	Y2	
Boys	0.84	0.85	0.77	0.85	⁎	0.86	0.87	0.73	0.83	⁎	0.83	0.96	⁎	0.78	0.79	
Girls	0.81	0.82	0.69	0.80	⁎	0.87	0.87	0.75	0.80	⁎	0.88	0.96	⁎	0.74	0.77	⁎
++			+	+												
*N*	8,802	9,769	15,725	13,401		3,751	5,626	2,065	3,371		2,296	2,625		8,264	22,243	

	Mongolia			Serbia		Sierra Leone		Swaziland			Guyana			Gambia		
	Y1	Y2		Y1	Y2	Y1	Y2	Y1	Y2		Y1	Y2		Y1	Y2	
Boys	0.87	0.92	⁎	0.92	0.89	0.83	0.81	0.86	0.89	⁎	0.79	0.90	⁎	0.44	0.75	⁎
Girls	0.90	0.94	⁎	0.90	0.87	0.81	0.82	0.84	0.88	⁎	0.80	0.90	⁎	0.45	0.76	⁎
++																
*N*	4,283	4,497		3,193	1,821	11,304	17,669	8,652	5,652		5,551	5,619		7,579	13,438	

Average	All	Y1	Y2
Boys	0.82	0.77	0.86
Girls	0.81	0.77	0.85

**(d) Work time (economic or domestic; average number of hours per week)**																
	Nigeria			Burundi			Cameroon			Togo		Côte d’Ivoire			Suriname	
	Y1	Y2	∗∗	Y1	Y2		Y1	Y2		Y1	Y2	Y1	Y2		Y1	Y2
Boys	9.35	7.34	⁎	23.0	19.3	⁎	26.80	15.11	⁎	11.04	11.75	15.61	11.62	⁎	1.03	0.31
Girls	9.45	9.38		23.5	17.3	⁎	31.22	15.69	⁎	14.30	16.68	24.71	16.49	⁎	1.30	0.31
++		+								+	+	+	+			
*N*	21,482	19,296		2,864	11,727		3,923	4,183		4,913	4,018	10,142	12,380		1,041	1,734

	Mongolia			Sierra Leone			Swaziland			Guyana		Vietnam			Gambia	
	Y1	Y2		Y1	Y2		Y1	Y2		Y1	Y2	Y1	Y2		Y1	Y2
Boys	16.41	5.27	⁎	17.76	8.35	⁎	8.18	2.30	⁎	6.74	6.95	6.29	2.50	⁎	6.33	5.68
Girls	15.64	3.65	⁎	18.11	8.90	⁎	7.93	2.81	⁎	6.32	6.53	9.54	4.07	⁎	6.43	9.97
++												+				+
*N*	905	1,083		6,594	7,425		1,713	1,108		1,749	2,272	28,475	3,171		6,705	11,284

Average	All	Y1	Y2
Boys	10.21	12.38	8.04
Girls	11.68	14.04	9.32

*Source:* Author’s calculation based on MICS data.

+ Indicates that the difference *between boys and girls* in each year is statistically significant (5%) (significance between rows).

⁎ Indicates that the difference *across periods* is statistically significant (5%) for boys or girls, respectively (significance between columns).

**Table 8:** Cross-tabulation for all indicators

**(a) Stunting and school attendance**				
	None	Bias for boys	Bias for girls	Total
None	0	17	31	48
Bias for boys	30	1,659	1,807	3,496
Bias for girls	21	666	751	1,438
Total	51	2,342	2,589	4,982

**(b) Stunting and working hours**				
	None	Bias for boys	Bias for girls	Total
None	0	40	6	46
Bias for boys	7	2,420	321	2,748
Bias for girls	2	984	160	1,146
Total	9	3,444	487	3,940

**(c) Birth registration and school attendance**				
	None	Bias for boys	Bias for girls	Total
None	0	45	56	101
Bias for boys	28	833	930	1,791
Bias for girls	60	2,435	2,758	5,253
Total	88	3,313	3,744	7,145

**(d) Birth registration and working hours**				
	None	Bias for boys	Bias for girls	Total
None	0	62	10	72
Bias for boys	6	1,199	186	1,391
Bias for girls	13	4,155	580	4,748
Total	19	5,416	776	6,211

**(e) School attendance and working hours**				
	None	Bias for boys	Bias for girls	Total
None	36	340	64	440
Bias for boys	85	9,374	1,662	11,121
Bias for girls	82	10,778	2,424	13,284
Total	203	20,492	4,150	24,845

*Source:* Author’s calculation based on MICS data.

**Table 9:** Cross-tabulation for all indicators by country

	Kazakhstan	Albania	Belize	Bosnia and Herzegovina	Burundi	Cameroon	Côte d’Ivoire	Gambia	Guyana	Lao PDR
Cramer’s *V*										
Stunting/birth registration	0.037	0.475	0.500	–		–	–	0.128	0.278	0.187
Stunting/school attendance	0.180	0.463	–0.033			–	–	0.018	0.058	0.026
Stunting/working hours	–	–	–	–		–	–	0.047	0.098	
Birth registration/school attendance	0.061	0.375	0.068	–	0.063	0.045	0.051	0.038	0.044	0.016
Birth registration/working hours	–	–	–	–	0.059	0.080	0.067	0.035	0.089	–
School attendance/working hours	–	–	–	–	0.021	0.099	0.030	0.033	0.205	–
*P* statistic for boys										
Stunting/birth registration.	0.893	0.698	0.859	–	–	–	–	0.721	0.821	0.623
Stunting/school attendance	0.836	1.000	0.818	–	–	–	–	0.766	0.807	0.629
Stunting/working hours	–	–	–	–	–	–	–	0.893	0.844	–
Birth registration/school attendance	0.513	0.000	0.529	–	0.420	0.473	0.442	0.423	0.519	0.486
Birth registration/working hours	–	–	–	–	0.930	0.883	0.849	0.888	0.711	–
School attendance/working hours	–	–	–	–	0.892	0.857	0.909	0.891	0.812	–
Average	0.747	0.566	0.736		0.747	0.738	0.733	0.764	0.752	0.580
*P* statistic for girls										
Stunting/birth registration	0.631	0.578	0.807	–	–	–	–	0.685	0.563	0.624
Stunting/school attendance	0.214	1.000	0.500	–	–	–	–	0.551	0.533	0.525
Stunting/working hours	–	–	–	–	–	–	–	0.294	0.364	–
Birth registration/school attendance	0.667	0.625	0.843	–	0.646	0.679	0.744	0.605	0.631	0.633
Birth registration/working hours	–	–	–	–	0.759	0.780	0.663	0.694	0.639	–
School attendance/working hours	–	–	–	–	0.530	0.630	0.559	0.537	0.634	–
Average	0.504	0.734	0.717		0.645	0.696	0.656	0.561	0.561	0.594

Absolute difference	0.24	0.17	0.02		0.10	0.04	0.08	0.20	0.19	0.01

	Mongolia	Nigeria	Serbia	Sierra Leone	Suriname	Swaziland	Togo	Trinidad and Tobago	Vietnam	Iraq
Cramer’s *V*										
Stunting/birth registration	0.233	0.080	0.149	0.180	0.224	0.098	0.150	–	–	0.194
Stunting/school attendance	0.124	0.055	0.167	0.083	0.049	0.038	0.058	–	–	–
Stunting/working hours	0.038	0.037	–	0.058	0.143	0.103	0.078	–	–	–
Birth registration/school attendance	0.135	0.006	0.123	0.055	0.071	0.030	0.035	0.149	0.082	–
Birth registration/working hours	0.183	0.024	–	0.018	0.127	0.080	0.084	–	0.016	–
School attendance/working hours	0.408	0.041	–	0.144	0.272	0.189	0.094	–	0.072	–
*P* statistic for boys										
Stunting/birth registration.	0.810	0.757	0.913	0.649	0.909	0.668	0.727	–	–	0.819
Stunting/school attendance	0.697	0.707	0.778	0.640	0.880	0.647	0.712	–	–	–
Stunting/working hours	0.929	0.888	–	0.879	0.931	0.911	0.862	–	–	–
Birth registration/school attendance	0.424	0.459	0.333	0.537	0.444	0.491	0.516	0.481	0.395	–
Birth registration/working hours	0.875	0.907	–	0.872	0.884	0.853	0.858	–	0.681	–
School attendance/working hours	0.866	0.849	–	0.875	0.938	0.955	0.876	–	0.757	–
Average	0.767	0.761	0.675	0.742	0.831	0.754	0.759	0.481	0.611	0.819
*P* statistic for girls										
Stunting/birth registration	0.522	0.909	0.696	0.725	0.697	0.665	0.692	–	–	0.589
Stunting/school attendance	0.500	0.548	0.429	0.506	0.524	0.445	0.500	–	–	–
Stunting/working hours	0.250	0.363	–	0.362	0.222	0.462	0.273	–	–	–
Birth registration/school attendance	0.538	0.884	0.759	0.742	0.676	0.676	0.699	0.649	0.589	–
Birth registration/working hours	0.250	0.916	–	0.741	0.700	0.615	0.636	–	0.598	–
School attendance/working hours	0.564	0.598	–	0.595	0.765	0.771	0.608	–	0.564	–
Average	0.437	0.703	0.628	0.612	0.597	0.606	0.568	0.649	0.584	0.589

Absolute difference	0.33	0.06	0.05	0.13	0.23	0.15	0.19	0.17	0.03	0.23

*Source:* Author’s calculation based on MICS data.

## Appendix 1 Indicators

The indicators and definitions follow as closely as possible those used by UNICEF for global reporting.

• Stunting: children under five years of age (0–59 months) whose height-for-age is below minus two standard deviations (moderate and severe) from the median height-for-age of the reference population.
• Birth registration: children less than five years of age (0–59 months) whose births were registered; that is, whose birth certificate was seen by the interviewer or whose mother or caretaker says the birth has been registered.
• School attendance: children between 6 and 15 years old attending primary school, secondary school, or a higher level.^21^

• Working hours: number of hours per week of economic work (paid or unpaid work outside the household), of domestic work (work in the family farm or business and/or inside the household), and of chores worked by children of between 6 and 15 years old.

## Appendix 2 Country sample

Population figures from: UN World Population Prospects, 2015.

^a^Figures are for 2000, 2005 and/or 2010 closest to the year of reference for each country.

## Appendix 3 Inequality decomposition

Tables 5(a)–(d).

## Appendix 4 Direction of the bias within households

Tables 6(a)–(d).

## Appendix 5 Summary statistics

Tables 7(a)–(d).

## Appendix 6 Cross-tabulations

Tables 8(a)–(d).

## Footnotes

1 Even if these are aggregate differences, rather than differences captured within households, they can be indicative of the type of parental preference for one gender over the other.

2 Developed at the Townsend Centre for International Poverty Research at the University of Bristol. It examined child deprivations in seven dimensions of well-being: shelter, sanitation, safe drinking water, information, food, education, and health.

3 Variables are recoded to match this interpretation.

4 This method is only able to capture whether some difference exists between boys and girls. Other methods (e.g., Alkire *et al.*, 2013) can be used to estimate the gap between both genders, but they make sense only for cardinal indicators (or composite multidimensional poverty measures).

5 At this point, the household level variables are cardinal but can be highly discontinuous, especially for smaller households. The discontinuity of the variable is unlikely to affect the mean value for each household, and thus the inequality measure.

6 On average, for all countries, the share of households kept in the analysis is 18% for stunting, 19% for birth registration, 38% for school attendance, and 20% for work time.

7 The average household size varies from 6 to 8 members in the sample of countries in this study.

8 There is no conceptual reason to believe that the household size would be correlated with intrahousehold gender bias. Moreover, empirical testing for this paper showed inconclusive results.

9 The final variable is cardinal, and thus differs from the alternative of assigning ordered numerical values to an ordinal variable (say 1, 2, 3 representing points in a happiness scale), which is sensitive to the scale used (see for example Dutta and Foster (2013)).

10 This procedure also reduces the discontinuity of the variables.

11 Apart from the within and between components, the Gini coefficient has a non-zero residual term and is not sub-group consistent.

12 Infant/child mortality represents the probability of dying before reaching a certain age.

13 For example, to analyse the joint distribution of stunting and birth registration, only households with data on both indicators are used. Given that some indicators are only relevant or collected for children in certain age ranges, this can considerably reduce the sample size with an increasing number of indicators.

14 All other cross-tabulations are presented in Appendix 6.

15 According to the World Bank classification.

16 There are also differences in the standards for measuring nutrition indicators, which largely depend on the underlying population reference group. Patterns differ substantially depending on whether the old National Center for Health Statistics (NCHS)/World Health Organization (WHO) standards or the more recent 2006 WHO standards are used: in particular, stunting is likely to be higher when using the new standards (de Onis, Onyango, Borghi, Garza, & Yang, 2006). The WHO standards are probably better at capturing the extent of malnutrition in a given country because their base population reference is a sample of breast-fed children selected from a wide geographical distribution. Nonetheless, the old NCHS/WHO population reference standards have been used in this study to compute stunting rates and the respective inequality indicators. This was done to ensure comparability over time, because MICS from round 2 and most of round 3 were conducted when this was the standard in place, and the data were reported accordingly.

17 The large jumps in between-household inequality in Albania, Gambia, and to a lesser degree in Burundi can be explained by the behaviour of extreme cases—that is, cases where no children are in school. In the second year, there is a large reduction in these cases, which can be a result of either progress in the dimension or measurement error at the time of collecting the initial survey.

18 These averages include girls and boys who do not engage in work or chores at all (zero hours a week). The average number of working hours is 19.8 for boys and 20.5 for girls.

19 The large jumps in between-household inequality in Burundi and Mongolia can be explained by the behaviour of extreme cases, that is, cases where children work zero hours. In the second year, there is a large increase in these cases, which can be a result of either progress in the dimension or measurement error at the time of collecting the survey.

20 Bosnia and Herzegovina has insufficient data to calculate pairings.
